# How Do Individually Ventilated Cages Affect the Welfare of Male BALB/c Mice? Comprehensive Assessment of Behavior, Metabolism, and Responses to Acute Painful Stimuli

**DOI:** 10.1002/brb3.70601

**Published:** 2025-05-30

**Authors:** Aslı Çelik, Cemre Ural, Hatice Efsun Kolatan, Pembe Keskinoğlu, Mehmet Ateş, Zahide Çavdar, Osman Yılmaz, Mehmet Ensari Güneli

**Affiliations:** ^1^ Vocational School of Health Services Dokuz Eylul University Izmir Turkey; ^2^ Multidisciplinary Experimental Animal Laboratory, Faculty of Medicine Dokuz Eylul University Izmir Turkey; ^3^ Department of Laboratory Animal Science, Health Sciences Institute Dokuz Eylul University Izmir Turkey; ^4^ Department of Molecular Medicine, Health Sciences Institute Dokuz Eylul University Izmir Turkey; ^5^ Department of Biostatistics and Informatics Dokuz Eylul University Medical School Izmir Turkey; ^6^ Biophotonics and Optical Imaging Laboratory Izmir Biomedicine and Genome Center Izmir Turkey

**Keywords:** anxiety levels, individually ventilated cage, open‐top cage, pain response behavior

## Abstract

**Introduction:**

Housing conditions, which have a major impact on the welfare of laboratory animals, are an important issue in experimental research. Individually ventilated cage (IVC) and open‐top cage (OTC) systems are widely used for housing laboratory mice.

**Purpose:**

This study aimed to comprehensively investigate the effects of OTC and IVC housing conditions on the behavior, metabolism, and pain responses of laboratory mice from an animal welfare perspective.

**Method:**

We measured body temperature, body weight, anxiety levels (using the elevated plus maze and open field test), and thermal nociceptive responses (using the hot‐plate and tail‐flick tests) in male albino BALB/c mice. At the end of these procedures, the mice were sacrificed, and the serum levels of adrenocorticotropic hormone (ACTH), corticosterone (CORT), ghrelin, and leptin were determined by ELISA, and the weight of the adrenal glands was measured.

**Findings:**

The results showed that there were significant differences in body weight, body temperature, anxiety‐related behaviors, pain latency, and hormonal parameters between the OTC group and the IVC group. Compared to OTC, IVC had lower levels of leptin, especially under stress conditions, where a significant interaction between housing and stress was observed, and higher levels of ghrelin, ACTH, and CORT. IVC group also had increased body weight, adrenal gland weight, and body temperature. In the hot‐plate test, the IVC group showed increased latency of hind limb responses compared to the OTC group, but not in the tail‐flick test. IVC group exhibited more anxiety‐related behaviors in the OFT, while no differences were observed in the EPM.

**Conclusion:**

According to the results of this study, housing mice in IVCs appears to compromise welfare, altering behavioral, hormonal, and pain responses. This suggests that the IVC system can induce physiological and behavioral stress, potentially acting as a systemic confounding factor in mouse research.

## Graphical abstract

IVC housing conditions cause significant changes in behavioral patterns, metabolism, and nociceptive responses in mice.

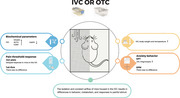

## 1 Introduction

The use of laboratory animals in scientific research requires a holistic approach to animal welfare, which includes maintaining their health and physiology, ensuring they can thrive in their living conditions, and allowing them to exhibit natural behaviors. The principles of the 3Rs—replacement, reduction, and refinement—introduced by Russell and Burch in 1959, are foundational for improving the welfare of animals used in experiments (Russell and Burch 1959). Rather than being strict rules applied sequentially, the 3Rs serve as guiding principles. Replacement encourages the use of alternative research methods before resorting to animal models. Reduction focuses on using the minimum number of animals necessary to achieve scientific objectives, while refinement involves enhancing experimental procedures to minimize pain and distress, and improving care and housing conditions for the animals (Baumans et al. 2002; Burman et al. 2014; Åhlgren and Voikar 2019).

The housing conditions of laboratory animals have a direct impact on their welfare, which can affect experimental results. It is not possible to prevent the animal's environment from affecting it. But, certain cage systems merely serve to try to control for these effects. Standardized cage systems are used to prevent these effects based on factors like genetic/microbiological status, biosafety levels, and research requirements. The most common cage systems are the open‐top cage (OTC) and the individually ventilated cage (IVC) (National Research Council (US) Committee for the Update of the Guide for the Care and Use of Laboratory Animals 2011; Spangenberg et al. 2014; Åhlgren and Voikar 2019).

The IVC system and the OTC system both have their advantages and disadvantages. The IVC system reduces humidity, carbon dioxide (CO2), ammonia levels, frequency of cage changes, and allergens in the environment, whereas the OTC system displays the opposite effects (Baumans et al. 2002). On the other hand, the OTC system has advantages despite its disadvantages. The IVC system experiences cold air flow and noise between cages, as well as vibrations due to the filtering system, which is not present in the OTC system. Moreover, stress is observed in animals in the IVC system as a result of decreased human‐animal interaction (Mineur and Crusio 2009; Kostomitsopoulos et al. 2012; Segelcke et al. 2023). As a result of these effects on cage systems, laboratory animals exhibit distinct behavioral and physiological responses.

Rodents regulate their body temperature through hormone release in the brain in response to environmental conditions, aiming to maintain a stable body temperature. Housing (macroenvironment) and cage (microenvironment) conditions should be standardized for rodents to maintain body temperature homeostasis (Gordon 1993; Gaskill et al. 2013). Housing differences can induce stress in experimental animals, leading to increased body temperature and altered body weight, metabolic hormone imbalances such as decreased leptin and increased ghrelin, and elevated levels of stress‐related hormones such as corticosterone (CORT) in mice (Gaskill et al. 2013; Haque et al. 2013; Itai et al. 2018; Abizaid 2019).

Animal behavior changes due to differences in housing conditions. Many studies analyze the effects of stress on animal behavior using a variety of tests. The elevated plus maze (EPM) and open field test (OFT) are commonly used behavioral assessments to measure stress levels in animals (Carola et al. 2002; Walf and Frye 2007; Gould 2009; Mineur and Crusio 2009). Housing variations affect the level of pain in mammals. Additionally, numerous factors contribute to varying pain levels within mammalian organisms, and stressors may impact pain levels for a variety of reasons. To understand how housing affects stress and consequently impacts pain‐related behavior, we measured the concentration of corticosterone metabolites in plasma (Takao et al. 2016; Oyola and Handa 2017; Pasquarelli et al. 2017; Segelcke et al. 2023). Therefore, important factors such as housing can cause stress in animals, which may affect behavioral and pain tests This study compared the behavioral, metabolic, and pain‐related responses of male BALB/c mice housed in OTC and IVC systems to evaluate the potential welfare implications.

## 2 Materials and Methods

### 2.1 Animals

The local ethics committee for animal experiments at Dokuz Eylul University Faculty of Medicine (DEUMF) approved this study (protocol number 08/2019).To the best of our abilities, the present animal study is reported in accordance with the so‐called ARRIVE guidelines (Kilkenny et al. 2010; du Sert et al. 2020).

Our study involved 48 male BALB/c albino mice aged 8–10 weeks (DEUMF, Multidisciplinary Experimental Animals Laboratory, Inciraltı, İzmir), housed in two microenvironmental conditions: OTC systems (1290D, Conventional, Tecniplast, Buguggiate, Italy) and IVC systems (1285L, Blue Line, Tecniplast, Buguggiate, Italy). The mice were housed in cages with four mice per cage, with ventilation provided through room air in OTC and a tower‐type blower in IVC.

Animals were fed irradiated pelleted diets (ARDEN, Research & Experimental Medical Materials, Ankara) and filtered tap water. Mice had ad libitum access to food and water throughout the study. Corn granules (ARDEN Research & Experimental Medical Materials, Ankara) were used as cage bedding. The study housed mice in a controlled environment with room temperature at 22 ± 2°C, relative humidity at 50 ± 10%, a 12‐h light‐dark cycle (lights on at 7:00 a.m.), red light for night vision, daytime light intensity of 200 lux, room‐level ventilation at 7–8 changes per hour, and a maximum noise level of 75 dB. The cage‐level ventilation for the IVCs was (smart flow, Tecniplast, Buguggiate, Italy) changes per hour. The OTC and IVC systems were kept separately to isolate them from laboratory traffic. Only male mice were housed, and physical contact was prohibited except for a veterinarian and animal caretaker. Mice were inspected daily, and their bedding and cage were changed weekly.

#### 2.1.1 Study Design

The study involved mice paired monogamously on P90, housed in an OTC and IVC system. The F1 generation was the offspring of dams with a 30‐day lactation period. After weaning, F2 generation mice were born, sexed at P30, and divided into cages. Cotton was used as cage enrichment and nest material during nursing. Only male mice of the F2 generation were included in the study at day 63. A total of 48 male mice were randomly assigned to four experimental groups: OTC naive control (OTC‐NG, *n* = 8), IVC naive control (IVC‐NG, *n* = 8), OTC experimental (OTC‐EG, *n* = 16), and IVC experimental (IVC‐EG, *n* = 16). The naive groups (OTC‐NG and IVC‐NG) were used for comparison with the experimental groups (OTC‐EG and IVC‐EG) to exclude the effects of behavioral tests (such as anxiety and pain). In the experimental groups, measurements included body weight, body temperature, behavioral tests, and blood hormone levels. In the naive groups, only body weight, body temperature, and serum tests were performed, with no behavioral tests. Mice were housed in 12 cages, with four mice per cage.

**FIGURE 1:**
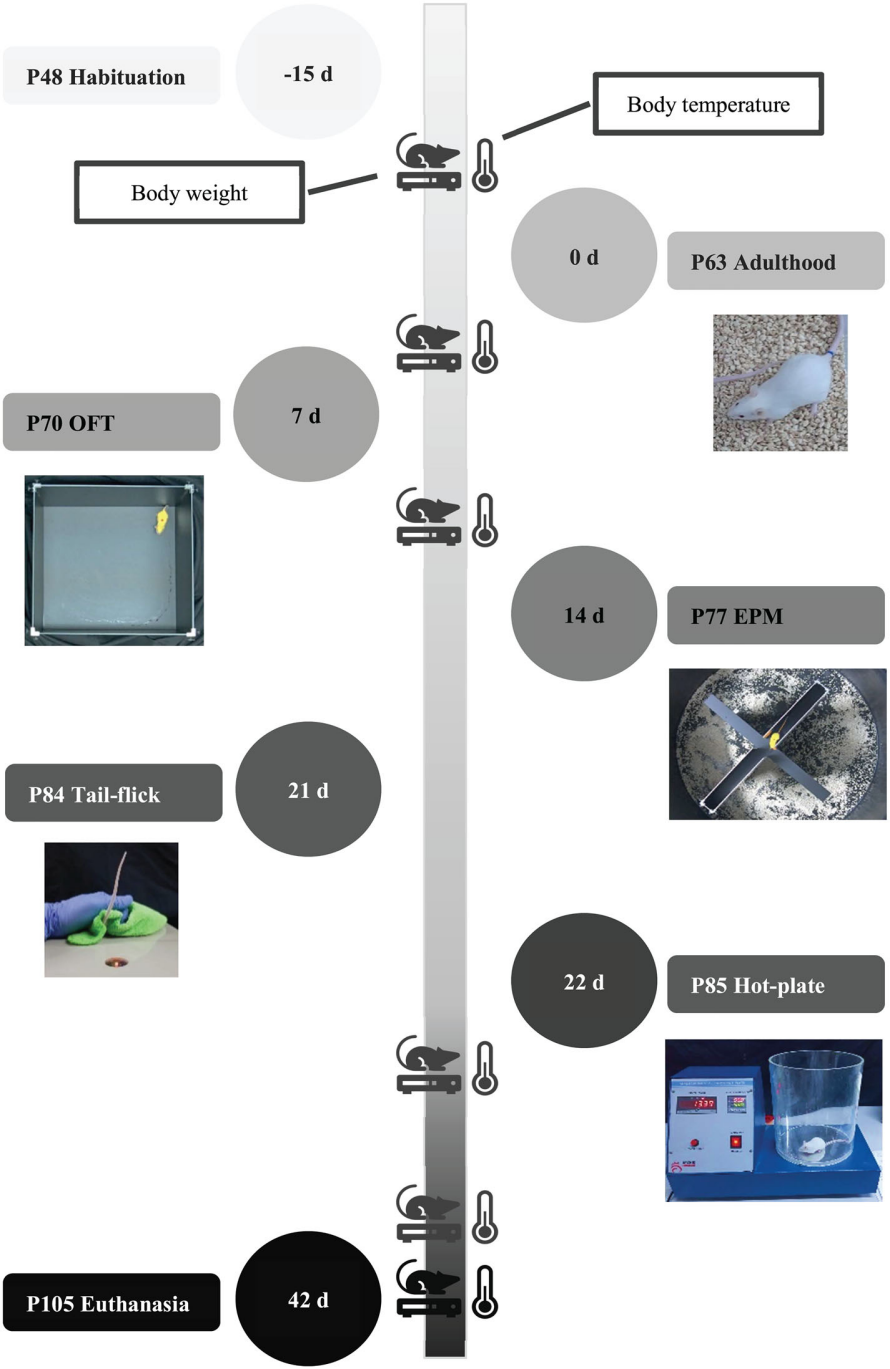
Experimental design (57 days). The body weight and temperature of the mice were measured at the ages of P58, P66, P76, P86, P103, and P105. d: day P: postnatal day, EPM: elevated plus maze, OFT: open field test, CORT: serum corticosterone.

Body weight and body temperature were measured regularly (between 9 and 12 a.m.) in all groups. Behavioral tests were designed according to Pasqurelli et al. (Pasquarelli et al. 2017). The flowchart of the experiment is shown in Figure 1. Equipment was disinfected with 70% ethyl alcohol after each test in all procedures.

### 2.2 Measurement of Body Temperature and Body Weight

The study involved four mice in each cage, separated for 5 min before returning to the cage to avoid impacting physiological, behavioral, or pain response results (Takao et al. 2016). Body weight was measured on days P30, P58, P66, P76, P86, P103, P105 and body temperature was measured on days P58, P66, P76, P86, P103 between 9 a.m. and 12 p.m. The temperature of each mouse was fixed before recording. Body weight was measured using a digital bench scale (Aeron, PT‐805) with a precision of 0.1 g. Each mouse was placed individually on the scale, and the weight was recorded. Body temperature was measured using a rectal probe (ATP Instrumentation Ltd., UK). The probe was gently inserted into the mouse's rectum to a standard depth, and the temperature was recorded once it stabilized. Care was taken to minimize stress during this procedure.

### 2.3 Behavioral Tests

Mice in the OTC‐EG and IVC‐EG were tested using the open field test (OFT) and elevated plus maze (EPM) at postnatal day 70 (P70) and postnatal day 77 (P77), respectively. Before testing, mice were acclimated to the behavioral testing area for 1 h. The behavioral testing room was maintained under controlled conditions to minimize confounding variables. The temperature was kept at 22 ± 2°C, humidity at 50 ± 10%, and a 12‐h light/dark cycle (lights on at 7:00 a.m.) was followed. For the OFT, the light intensity was set at 90 lux, while for the EPM, the light intensity was set at 65 lux. All tests were conducted between 9:00 a.m. and 12:00 p.m. to reduce circadian variability.

In the OFT, the center area of the test arena was used to assess anxiety‐related behaviors. Anxiety is indicated by a mouse's tendency to spend less time in the center of the arena and more time near the walls, known as the thigmotaxis behavior. This behavior suggests an avoidance of open spaces, which is characteristic of anxiety‐related responses (Gould et al. 2009; Burman et al. 2014).

For the EPM, mice were placed at the junction of the two open and two closed arms, facing the open arm at the start of the test. The amount of time spent in the open arms versus the closed arms inferred anxiety. A higher amount of time spent in the closed arms is indicative of higher anxiety levels, while spending more time in the open arms suggests lower anxiety levels (Mineur and Crusio 2009; Burman et al. 2014; Pasquarelli et al. 2017). Each test lasted 5 min, during which time defecation incidents were recorded as an indicator of anxiety. Increased defecation has been widely recognized as a physiological marker of anxiety and stress in rodents (Gould 2009; Burman et al. 2014). The OFT and EPM are well‐established behavioral assays for measuring anxiety in rodents. The OFT assesses anxiety‐related behaviors by measuring the balance between exploration of a novel environment (center of the arena) and the tendency to stay in safer, enclosed areas (periphery of the arena), which is a common behavioral sign of anxiety in animals. The EPM evaluates anxiety based on the rodent's natural avoidance of open spaces, which simulates a fear of heights and open areas, commonly associated with anxious states in animals. Both tests are widely used and have been validated as reliable measures of anxiety (Mineur and Crusio 2009; Burman et al. 2014; Pasquarelli et al. 2017).

#### 2.3.1 Open Field Test (OFT)

The OFT was utilized to study locomotor activity and thigmotaxis in mice. The open test area was 45×45×45 cm and divided into 25 equal areas. Each mouse was placed in the center of the arena and allowed to explore for 5 min. The activity was monitored using an overhead camera and analyzed using specialized video tracking software (Ethovision XT 13, Noldus, The Netherlands)(Mineur and Crusio 2009; Burman et al. 2014; Kinlein et al. 2019). The parameters evaluated included frequency of entry to inner and outer zones, time spent in these areas, frequency, total distance moved, speed, and total time spent moving and resting during the test period (Gould et al. 2009; Pasquarelli et al. 2017).

#### 2.3.2 Elevated Plus Maze (EPM)

A plus‐shaped maze consisting of two open and two closed arms was used. The test was conducted in an experimental setup 40 cm above the floor, with two open and two closed arms of 38×6 cm, and the height of the closed arms was 15 cm. The inner surfaces of the closed arms, the floor of the open arms, and the floor of the open arms were matte black, while the outer surface of the closed arms was glossy white. The EPM test arena was placed in an empty, rat‐specific morris water maze tank to minimize the risk of mice falling from open arms. Open and closed arms were placed 24 cm from the water tank, and the gradient was adjusted to zero using a spirit level. (Bubble level, mobile app, Gamma Play). Homogeneous light distribution was ensured by measuring light intensity with a lux light meter (lux light meter pro, mobile app, Doggo Apps) (Gould 2009). Mice were placed individually in the center, facing the same open arms, and a camera observed anxiety‐related behaviors for 5 min. Analyses were performed using special video monitoring software (Ethovision XT 13, Noldus, The Netherlands) (Carola et al. 2002; Walf and Frye 2007; Pasquarelli et al. 2017). The parameters evaluated in EPM were the number of entries into the inner zone, the open and closed arms, and mouse duration in these areas (Carola et al. 2002).

### 2.4 Nociceptive Tests

The study used tail‐flick and hot‐plate nociception tests to measure the pain threshold of mice in different cage systems. Tail‐flick tests are determinant tests of spinal reflex responses, while hot‐plate tests show animals respond with higher structures like the central nervous system (Eddy and Leimbach 1953; Gregory et al. 2013; D'amour and Smith 1941). Mice were subjected to both tests at different times to avoid bias (Casarrubea et al. 2011; Deuis et al. 2017). The tests were repeated three times at 10‐min intervals, with a cut‐off time of 15 s for tail‐flick and 30 s for hot‐plate (Guneli, Kazikdas et al. 2007).

The tests were conducted in a controlled evaluation room where temperature was kept at 22 ± 2°C, humidity at 50 ± 10%, and a 12‐h light/dark cycle (lights on at 7:00 a.m.) was followed. For the tail‐flick and hot‐plate tests, the environmental light intensity was maintained at 150 lux and the tests were performed between 9:00 a.m. and 12:00 p.m. to reduce circadian variability.

#### 2.4.1 Tail‐Flick Test

The tail‐flick test was conducted using a radiant heat source focused on the tail's dorsal surface. Mice were examined for latency (seconds) to withdraw their tails from a noxious thermal stimulus using a tail‐flick meter (MAY‐TF 0703, Ankara, Turkey). The withdrawal of the tail exposed to the light turned off the thermal stimulus and automatically stopped the clock (Guneli, Kazikdas et al. 2007).

#### 2.4.2 Hot‐Plate Test

The nociceptive responses of mice were measured on a hot‐plate (Orchid Scientific and Innovative India Pvt. Ltd., Maharashtra, India) enclosed in a Plexiglas cylinder (Eddy and Leimbach 1953). The plate temperature was measured at 55°C. This temperature monitored painful responses without compromising animal welfare (Deuis et al. 2017). Response time to painful stimuli was determined as the onset of hind limb licking, withdrawal, or escape by jumping off the plate (Casarrubea et al. 2011).

### 2.5 Blood and Adrenal Gland Collection

At P105, all groups of mice were euthanized by exsanguination while under general anesthesia. Anesthesia was administered via an intraperitoneal injection of ketamine (100 mg/kg, Ketasol 10% Richter Pharma Ag, Austria) and xylazine (10 mg/kg, Xylazinbio 2% Bioveta, Czech Republic). Blood was collected from the caudal vena cava using an insulin injector. The collected blood was stored at room temperature for 1 h to allow clotting, then transferred to Eppendorf tubes and centrifuged at 3500 rpm for 10 min. The serum samples were separated, transferred to new Eppendorf tubes, and stored at −80°C for biochemical analysis by enzyme‐linked immunosorbent assay (ELISA) (Holubová et al. 2016).

The right and left adrenal glands were carefully removed from the mice. To ensure consistency, the adrenal glands were excised by making a midline incision to expose the abdominal cavity and locating the glands. Surrounding fat was meticulously removed using fine forceps under a dissecting microscope. The weights of the adrenal glands were measured using a balance (Kern abt 220–5dm, Germany).

### 2.6 Enzyme‐Linked Immunosorbent Assay (ELISA)

Leptin, ghrelin, ACTH, and CORT were analyzed by ELISA after serum from mice removed from the freezer was brought to room temperature. The serum sample of each mouse was divided into four parts.

The ELISA test is based on the antigen‐antibody relationship, and the amounts of antibody‐enzyme conjugates are determined through spectrophotometric measurement. The tests were performed according to the supplier's procedure. Serum leptin concentrations were determined by Mouse Leptin ELISA (Sunred Technology Co., Ltd., Shanghai, China), cat. no. 201‐02‐0651, and values were expressed as ng/mL. Ghrelin levels were determined using the rat/mouse ghrelin (active) kit (Millipore, USA), cat. no. EZRGRA‐90K, and values were expressed as pg/mL. ACTH levels were determined using a mouse ACTH ELISA kit (Sunred Technology Co., Ltd., Shanghai, China), cat. no. 201‐02‐0266, and values were expressed as pg/mL. CORT levels were determined using the Corticosterone Parameter Assay Kit (R&D Systems, Minneapolis, USA), cat. no KGE009, and values were expressed as ng/mL (Holubová et al. 2016). The results were analyzed using a reference standard gradient plot provided with the test material.

### 2.7 Statistical Analysis

A comparison of the parameters of a total of four groups, NG (*n* = 8) and EG (*n* = 16), of animals housed in OTC and IVC systems is shown in Table 1.

**TABLE 1:** Comparison table of parameters in the NG and EG.

Groups	OTC naive group (control) (OTC‐NG)	IVC experimental group (IVC‐EG)
OTC experimental group (OTC‐EG)	(i)	(ii)
IVC naive group (control) (IVC‐NG)	(iii)	(i)
(i) Evaluation of effect by procedure: change of environment—manipulation (handling, placement, time of exposure to multiple persons);—effect of possible unknown variables. (ii) Multiple habitat effects: behavior (OFT, EPM), pain (tail‐flick, hot‐plate), biochemistry (leptin, ghrelin, ACTH, KORT), physiology (body weight, body temperature, adrenal weights). (iii) Various effects of habitat: micro‐ and macro‐environmental effects, biochemistry (leptin, ghrelin, ACTH, KORT), physiology (body weight, body temperature, adrenal weights). (Adrenal weight data are shown in Table S3).		

All statistical analyses were performed using IBM SPSS Statistics (version 24). Data were preanalyzed for normality using the Shapiro‐Wilk test and homogeneity of variances using Levene's test. Depending on the distribution and variance characteristics, appropriate parametric or non‐parametric tests were applied.

For repeated measures data (e.g., body weight, body temperature, nociceptive latency), a repeated measures two‐way analysis of variance (RM‐ANOVA) was conducted to assess the effects of cage system (IVC vs. OTC) and group (EG vs. NG) over time. Pairwise comparisons with Bonferroni correction were performed to adjust for multiple testing when significant main effects or interactions were confirmed.

For behavioral assays with single‐time‐point outcomes (e.g., OFT, EPM), group comparisons were made using either independent samples t‐tests (for normally distributed variables) or the Mann–Whitney U test (for non‐normally distributed data). Behavioral parameters such as number of entries, time spent in zones, latency to entry, and locomotor variables were analyzed separately for each relevant area (e.g., inner zone, outer zone, corner zone).

For the ELISA hormone analyses, a two‐way ANOVA was used to evaluate the effects of cage system and group, and their interaction, on serum levels of ACTH, CORT, leptin, and ghrelin. Pairwise comparisons with Bonferroni correction were performed to adjust for multiple testing when significant main effects or interactions were confirmed.

## 3 Results

### 3.1 Body Weight

A repeated measure two‐way analysis of variance (RM‐ANOVA) was conducted to examine the effects of cage system (IVC vs. OTC) and group (EG vs. NG) on body weight changes across seven‐time points (P30, P58, P66, P76, P86, P103, P105). Significant main effects were observed for both the cage system and group, while the interaction between cage system and group was not significant. Cage system (IVC vs. OTC): F_1, 44_ = 232.228, *p* < 0.001; group type (EG vs. NG): F_1, 44_ = 13.260, *p* < 0.001; interaction (cage system * group type): F_1, 44_ = 0.028, *p* = 0.867.

**FIGURE 2:**
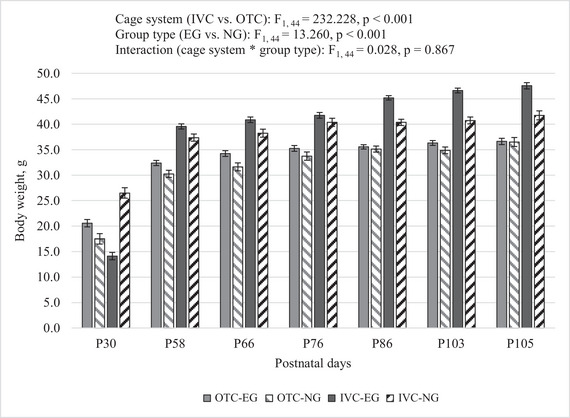
Body weight of mice during the study. Data represent group means of weight measurements taken at seven time points (P30, P58, P66, P76, P86, P103, P105). Error bars represent SEM. The number of mice in each group were as follows: OTC‐EG (*n* = 16), OTC‐NG (*n* = 8), IVC‐EG (*n* = 16), IVC‐NG (*n* = 8). We used RM ANOVA with a two factorial design to analyze how the cage system (IVC vs. OTC) and group (NG vs. EG) affected the results. A post‐hoc Bonferroni comparison was applied to determine significant differences between time points. At P30, at each time point that followed, the body weight of the mice was significantly less. The body weight of the mice was significantly less at P30 than at the other six measurement times, all with *p*‐values less than 0.001. Comparing P58 to P66, a small but significant difference was observed (*p* = 0.048). However, when body weight at P58 was compared to all subsequent time points (P66, P76, P86, P86, P103, and P105), mice were found to have less body weight than at each time point; *p* < 0.001 at each time point. The body weight measurements of mice were found to be consistently lower in P66 compared to P76, P86, P103, and P105. The *p*‐values were *p* = 0.004 and the others *p* < 0.001, respectively. Measurements of the body weight of mice were consistently lower in P76 compared to P86, P103, and P105; *p*‐values were *p* = 0.004 and others *p* < 0.001, respectively. Body weight measurements of mice at P86 showed no difference between P103 (*p* = 0.299) and were found to be lower (*p* < 0.001) compared to the P105 measurement.

Pairwise comparisons were conducted to examine the differences in body weight between the seven time points. The comparisons were adjusted for multiple comparisons using the Bonferroni correction. Significant differences were observed between all time points, with the greatest changes in body weight occurring between the initial time points. The progression of the body weight of the mice over time is shown in Figure 2. The overall pattern showed a gradual increase in body weight, with significant differences between several time points, particularly between the early and later stages of the study.

These findings indicate that both the cage system and the group type independently contribute to differences in weight changes over time, while the interaction between these factors is not significant.

### 3.2 Body Temperature

We found that mice housed in different cage systems had different body temperature scores, with IVC‐NG mice having higher body temperatures than OTC‐NG mice at five time points (see Table S2).

**FIGURE 3:**
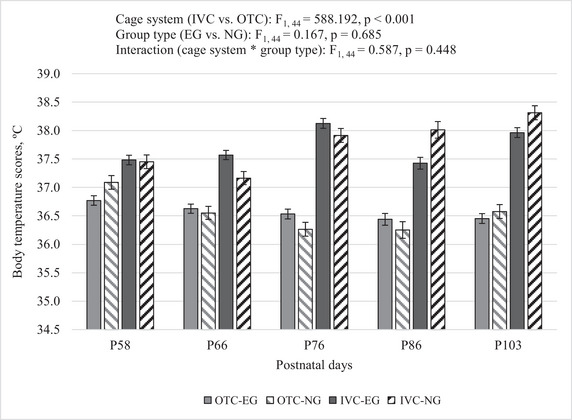
Body temperature of mice during the study. Data represent averages of body temperature measurements taken at five time points (P58, P66, P76, P86, P103). Error bars represent SEM. The number of mice in each group was as follows: OTC‐EG (*n* = 16), OTC‐NG (*n* = 8), IVC‐EG (*n* = 16), IVC‐NG (*n* = 8). We used RM ANOVA with a two factorial design to analyze how the cage system (IVC vs. OTC) and group (NG vs. EG) affected the results. A post‐hoc Bonferroni comparison was applied to determine significant differences between time points. A significant increase in body temperature was found at P58 compared to P66 (*p* = 0.028). There were no significant differences between measurements performed at P58 and P76, P86, and P103 (*p* > 0.05). There were no significant differences between the time points P66 and P76 and P86, whereas there was a lower body temperature at time point P103 (*p* < 0.001). There were no significant differences between the P76 measurement and the measurements performed at P86 and P103 times (*p* > 0.05). The body temperature measurement at P86 was significantly decreased in comparison to the measurement at P103 (*p* = 0.004).

A two‐way analysis of variance (RM‐ANOVA) with repeated measures was performed to examine the effects of cage system (IVC vs. OTC) and group (EG vs. NG) on the body temperature changes of mice at five time points (P58, P66, P76, P86, P103). Significant main effects were observed for the cage system (IVC vs. OTC). In contrast, significant main effects were observed for groups (EG vs. NG), and the interaction between the cage system and the groups was not significant. Cage system (IVC vs. OTC): F_1, 44_ = 588.192, *p* < 0.001; group type (EG vs. NG): F_1, 44_ = 0.167, *p* = 0.685; interaction (cage system * group type): F_1, 44_ = 0.587, *p* = 0.448. Mice housed in the IVC system exhibited significantly different body temperature changes compared to those housed in the OTC system. The progression of the body temperature of the mice over time is shown in Figure 3.

The significant differences in body temperature primarily occurred between the time points P66 and P103, with a notable increase in body temperature from P66 to P103. Specifically, body temperature was significantly higher at P66 compared to P58 and significantly lower at P103 compared to P66 and P86. No significant differences were observed between P58, P76, and P86 in most comparisons.

### 3.3 Open Field Test

**FIGURE 4:**
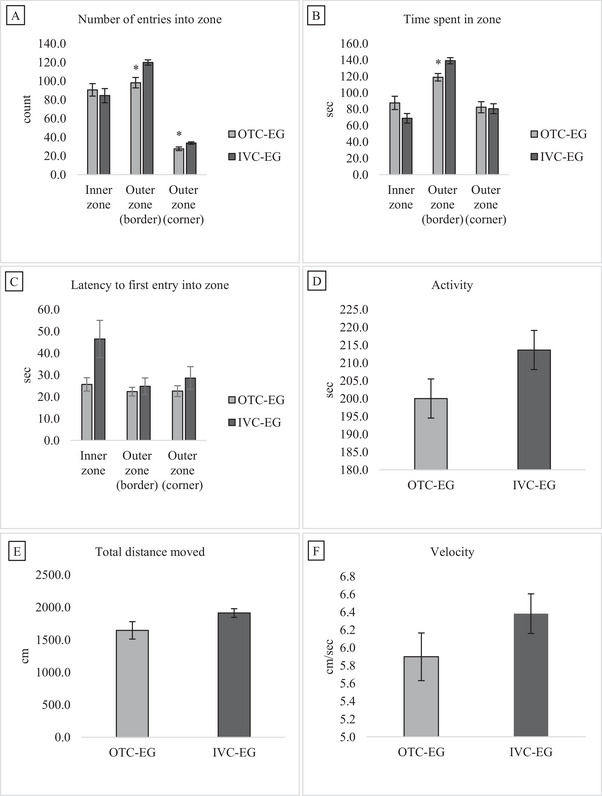
Comparison of the OFT area behavior of mice in the OTC‐EG and IVC‐EG groups. Data are presented as mean ± SEM for *n* = 16 mice per group. Data were not normally distributed; thus, the statistical comparisons were made using the non‐parametric Mann‐Whitney U test. The significance between groups is shown as *p < 0.01. (A) The number of entries into the zone of the outer zone of IVC‐EG was higher than that of OTC‐EG. There was no difference in the inner zone number of entries between groups (*p* > 0.05). (B) The time spent in the outer zone (border) of the IVC‐EG was found to be longer than in the OTC‐EG. There was no significant difference in the time spent in the inner zone and corner areas between the groups (*p* > 0.05). (C) latency to first entry into the zone, (D) total activity, (E) total distance moved, and (F) velocity were not significantly different (*p* > 0.05) between the two groups.

Figure 4 presents data on the number of entries into the zone, time spent in the zone, latency to first entry into the zone, velocity, and total duration of movement in the OFT areas of mice in the OTC‐EG and IVC‐EG groups. The data for each measure were analyzed using the non‐parametric Mann‐Whitney U test, as the data were not normally distributed. A significant difference was observed between groups for the number of entries into the outer zone (border) of the OFT. The IVC‐EG group made more entries into this zone compared to the OTC‐EG group (U = 43.50, *p* = 0.001). However, no significant difference was found in the number of entries into the inner zone (U = 114.50, *p* = 0.611) or the outer zone (corner) (U = 64.00, *p* = 0.01). The IVC‐EG group spent significantly more time in the outer zone (border) than the OTC‐EG group (U = 50.00, *p* = 0.003). No significant difference was observed in the time spent in the inner zone (U = 89.00, *p* = 0.142) or the outer zone (corner) (U = 126.00, *p* = 0.940). There was no significant difference in the latency to first entry into the inner zone (U = 83.50, *p* = 0.093), outer zone (border) (U = 126.50, *p* = 0.955), or outer zone (corner) (U = 121.00, *p* = 0.792). No significant differences were observed between the groups in total activity (U = 95.00, *p* = 0.214), total distance moved (U = 86.00, *p* = 0.113), or velocity (U = 76.50, *p* = 0.052).

### 3.4 Elevated Plus Maze

**FIGURE 5:**
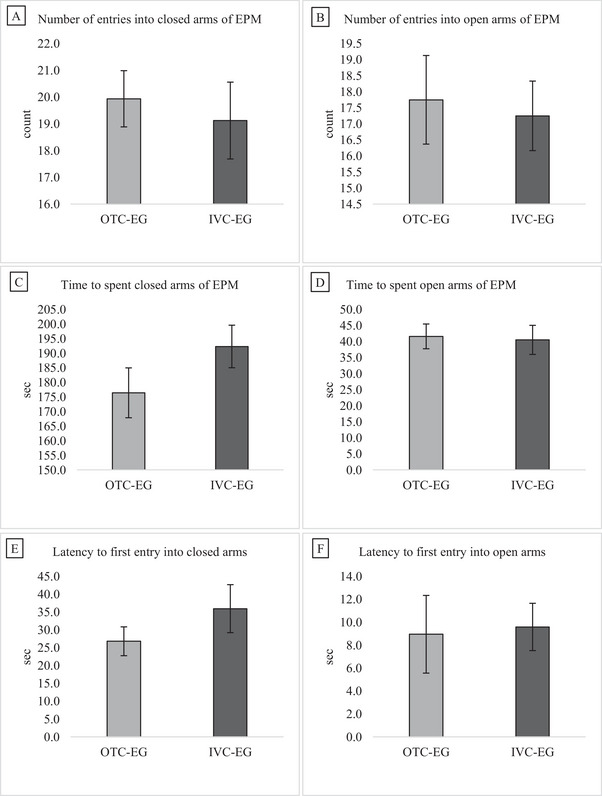
Comparison of the EPM area behavior of mice in the OTC‐EG and IVC‐EG groups. (A, B) The number of entries into the closed and open arms. (C, D) The time spent in the closed and open arms. (E, F) Latency to first entry into the closed and open arms. Data are presented as mean ± SEM for *n* = 16 mice per group. Statistical analysis was performed using the independent t‐test. There were no differences in the EPM area between groups (*p* > 0.05).

Figure 5 shows the number of entries into the zone, time spent in the zone, and latency to the first entry into the zone in the EPM area of mice in the experimental group housed in the OTC and IVC. Independent samples t‐tests were conducted to compare the two housing conditions (OTC and IVC) for each measured parameter. There was no significant difference between the two groups for the number of entries into the open arms (t(30) = 0.285, *p* = 0.389). The number of entries into the closed arms also showed no significant difference between groups (t(30) = 0.456, *p* = 0.326). Similarly, the time spent in the open arms did not differ significantly between the groups (t(30) = 0.183, *p* = 0.428). In contrast, there was a trend towards a significant difference in the time spent in the closed arms (t(30) = −1.416, *p* = 0.084). The latency to the first entry into the open arms did not differ significantly between the groups (t(30) = −0.160, *p* = 0.437). The latency to the first entry into the closed arms also did not show a significant difference (t(30) = −1.161, *p* = 0.127).

### 3.5 Number of Fecal Boli

The number of fecal boli was recorded during the OFT and EPM to assess anxiety‐related behavior. Statistical analysis was performed using the Mann‐Whitney U test for both tests. The data for each group consisted of *n* = 16 mice. In the OFT, no significant difference in the number of fecal boli was observed between the experimental groups (U = 86.50, *p* = 0.119). In the EPM, there was also no significant difference in the number of fecal boli between the groups (U = 117.00, *p* = 0.696) (see Figure S8).

### 3.6 Tail‐flick Test

Tail‐flick tests were performed at 0, 10, and 20 min on experimental groups composed of P84 mice to assess nociceptive responses. Repeated measures ANOVA revealed that housing conditions did not have a statistically significant effect on tail‐flick latency (F₁,₃₀ = 4.151, p = 0.051), although a trend toward significance was observed (see Figure S9).

Time‐dependent measurements indicated significant within‐group changes in nociceptive responses (F₁,₃₀ = 595.788, *p* < 0.001). In the OTC‐EG group, a significant increase in latency was observed between 0 and 20 min (mean difference = 2.437, p = 0.031), while differences between 0 and 10 min (mean difference = 1.375, *p* = 0.298) and between 10 and 20 min (mean difference = 1.062, *p* = 0.948) were not statistically significant.

In the IVC‐EG group, response latencies significantly increased from 0 to 10 min (mean difference = 2.625, *p* = 0.009) and from 0 to 20 min (mean difference = 3.812, *p* < 0.001). However, the increase from 10 to 20 min was not significant (mean difference = 1.188, *p* = 0.660).

### 3.7 Hot‐Plate Test

**FIGURE 6:**
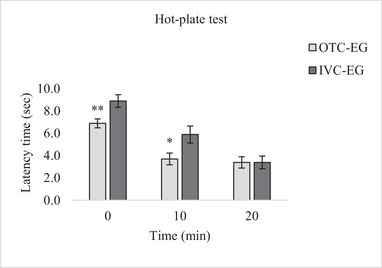
Hot plate test responses of mice in the experimental groups. Data are presented as mean ± SEM for *n* = 16 mice per group. Mice housed in IVC systems had a significantly longer hindlimb response at 0 and 10 min, while there was no significant difference at 20 min (*p* > 0.05). Statistical analysis was performed using the RM ANOVA test (pairwise comparisons using Bonferroni). Significant differences between groups were described as *p < 0.05 and **p < 0.01.

Hot‐plate tests were conducted at 0, 10, and 20 min in experimental groups consisting of P85 mice. Housing conditions significantly influenced nociceptive responses, as revealed by RM ANOVA. A significant main effect of housing condition was observed on pain responses (F₁,₃₀ = 9.726, *p* = 0.04). Further pairwise comparisons using Bonferroni correction demonstrated that the IVC group exhibited a delayed response to thermal stimuli compared to other groups. Specifically, significant differences were observed between groups at each time point: at 0 min, the mean difference was 2.000 (*p* = 0.007); at 10 min, 2.188 (*p* = 0.025); and at 20 min, no significant difference was found (mean difference = 0.000, *p* = 1.000) (Figure 6).

Time‐dependent measurements also revealed significant within‐group changes in nociceptive responses (F₁,₃₀ = 570.206, *p* < 0.001). In the OTC‐EG group, pairwise comparisons showed significant increases in latency from 0 to 10 min (mean difference = 3.188, *p* = 0.003) and from 0 to 20 min (mean difference = 3.500, *p* < 0.001), while no significant difference was found between 10 and 20 min (mean difference = 0.313, *p* = 1.000). Similarly, in the IVC‐EG group, latencies increased significantly over time: from 0 to 10 min (mean difference = 3.000, *p* = 0.005), 0 to 20 min (mean difference = 5.500, *p* < 0.001), and 10 to 20 min (mean difference = 2.500, *p* = 0.004).

These results suggest that mice in the IVC group had a delayed response to the thermal stimulus, as shown by the significant differences at 0 and 10 min, while there was no significant difference at 20 min.

### 3.8 ELISA Results

**FIGURE 7:**
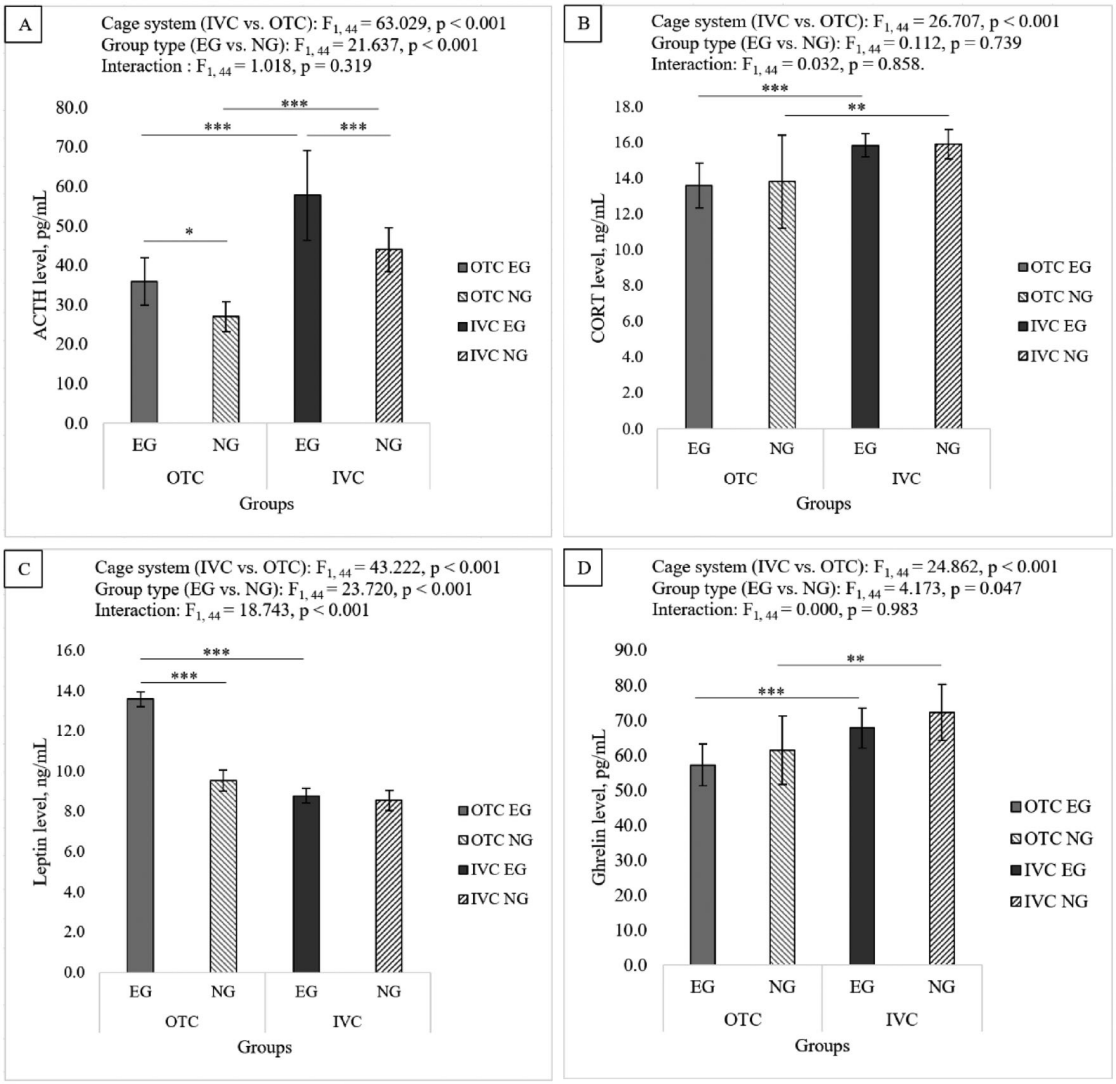
Comparison of ACTH, CORT, leptin, and ghrelin levels across groups. The number of subjects in each group was as follows: OTC‐NG (*n* = 8), OTC‐EG (*n* = 16), IVC‐NG (*n* = 8), and IVC‐EG (*n* = 16). Statistical analysis was performed using the two‐way ANOVA test. A Bonferroni adjustment was used to compare interactions between cage systems and groups. Significant differences between groups were described as *p < 0.05, **p < 0.01, and ***p < 0.001. Data are presented as means ± SEM. (A) Both the cage system and the group showed significant main effects for ACTH levels, but the interaction between the cage system and the group was not significant. Cage system (IVC vs. OTC): F_1, 44_ = 63.029, *p* < 0.001; group type (EG vs. NG): F_1, 44_ = 21.637, *p* < 0.001; interaction (cage system * group type): F_1, 44_ = 1.018, *p* = 0.319. (B) For CORT levels, significant main effects were observed for the cage system but not for the group, and the interaction between the cage system and the group was not significant. Cage system (IVC vs. OTC): F_1, 44_ = 26.707, *p* < 0.001; group type (EG vs. NG): F_1, 44_ = 0.112, *p* = 0.739; interaction (cage system * group type): F_1, 44_ = 0.032, *p* = 0.858. (C) For leptin levels, the interactions between cage system, group type, and cage system vs. group were all significant. Cage system (IVC vs. OTC): F_1, 44_ = 43.222, *p* < 0.001; group type (EG vs. NG): F_1, 44_ = 23.720, *p* < 0.001; interaction (cage system * group type): F_1, 44_ = 18.743, *p* < 0.001. (D) For ghrelin levels, there were significant differences in the cage system effect and group type, while the interaction between the cage system and group was insignificant. Cage system (IVC vs. OTC): F_1, 44_ = 24.862, *p* < 0.001; group type (EG vs. NG): F_1, 44_ = 4.173, *p* = 0.047; interaction (cage system * group type): F_1, 44_ = 0.000, *p* = 0.983.

Housing and procedures in different cages significantly affected the hormonal status of the mice. ELISA kits were used to measure ACTH, CORT, leptin, and ghrelin serum levels. Two‐way ANOVA analysis was performed to examine the effects of cage system (IVC vs. OTC) and group (EG vs. NG) on changes in serum hormone levels. Pairwise comparisons were performed using Bonferroni (Figure 7).

ACTH levels showed a significant effect of the cage system (*p* < 0.001) as well as a significant effect of the group type (*p* < 0.001). However, we observed no significant interaction between the cage system and group type (*p* = 0.319). Subsequent pairwise comparisons revealed that for the OTC cage system, OTC‐EG had significantly higher ACTH levels than OTC‐NG (*p* = 0.013). Similarly, IVC‐EG exhibited significantly higher ACTH levels for the IVC cage system than IVC‐NG (*p* < 0.001). For the different cage systems, the IVC‐EG group had significantly higher ACTH levels than the OTC‐EG group (*p* < 0.001), and the IVC‐NG group had significantly higher ACTH levels than the OTC‐NG group (*p* < 0.001) (Figure 7A).

In CORT levels, there was a significant effect of the cage system (*p* < 0.001) but not of the group type (*p* = 0.739), and there was no significant interaction between the cage system and the group type (*p* = 0.858). According to the different cage systems, the IVC‐EG group was significantly higher than the OTC‐EG group (*p* < 0.001), and the IVC‐NG group was significantly higher than the OTC‐NG group (*p* < 0.01) (Figure 7B).

Leptin levels showed a significant effect of the cage system (p < 0.001) and a group type (*p* < 0.001). A significant interaction between cage system and group type was observed (*p* < 0.001). Thereafter, pairwise comparisons revealed that OTC‐EG had significantly higher leptin levels than OTC‐NG (*p* < 0.001), but there was no significant difference between IVC‐EG and IVC‐NG (*p* = 0.704) in the IVC cage type. Furthermore, the OTC‐EG group had higher leptin levels than the IVC‐EG group (*p* < 0.001) (Figure 7C).

The cage system had a significant effect on ghrelin levels (*p* < 0.001), and group type also had a marginally significant effect (*p* = 0.047). However, the cage system and group type did not significantly interact (*p* = 0.983). IVC‐EG had significantly higher ghrelin levels than OTC‐EG, and IVC‐NG had significantly higher ghrelin levels than OTC‐NG (*p* < 0.001, *p* < 0.01, respectively) (Figure 7D).

ACTH levels were significantly affected by the cage system and group type, with EG having higher levels than NG in both cage systems (OTC and IVC). The cage system primarily influenced CORT levels, while group type had no significant effect. Leptin levels demonstrated a significant interaction between the cage system and group type, with EG exhibiting higher levels in the OTC system. Ghrelin levels were influenced by the cage system, with NG levels slightly higher than EG, but there was no significant interaction between the cage system and group type. All hormonal levels measured in serum were found to be influenced by the different cage systems of IVC and OTC.

## 4 Discussion

The main findings of this study were that the two cage systems used to house mice (IVC and OTC) showed different levels of variability in the behavioral, metabolic, and pain responses of the animals. Overall, we found that the cage environment affected body weight, body temperature, anxiety‐related behaviors, pain responses, and metabolic hormones. In mice housed in IVC cages: time‐dependent weight gain and body temperature increase; behavioral tests showed partly increased anxiety levels (tigmotaxis) and pain sensitivity in the hot plate test; increased stress markers such as ACTH, CORT, and adrenal gland weight; and metabolically increased ghrelin levels and decreased leptin levels. These findings highlight the importance of housing conditions in animal research in terms of animal welfare and their potential to influence experimental outcomes.

The widespread use of IVC for modeling behavioral and pain studies offers advantages such as suitability for laboratory standards (Gregory et al. 2013; Spangenberg et al. 2014; Deuis et al. 2017). Mice bred to laboratory standards are selected according to their characteristics, such as breed, sex, body weight, age, and experimental design (National Research Council (US) Committee for the Update of the Guide for the Care and Use of Laboratory Animals 2011). Different housing conditions, such as OTC and IVC, have a primary influence on the welfare of these animals (Baumans et al. 2002).

In our study, we examined changes in body weight in EG and NG mice housed in two different systems (OTC and IVC). Body weight measurements revealed that mice housed in IVC consistently gained more weight than OTC, with significant main effects for both cage system and group but no interaction. These findings demonstrate the influence of macroenvironment and microenvironment on the increased body weight gain of IVC compared to OTC. EG mice gained more weight than NG mice, suggesting that stress from the procedure influences body weight gain. Memarzadeh et al. found lower water intake and weight gain in mice housed in OTC compared to those housed in IVC (Memarzadeh et al., 2004), while Pasquarelli et al. found significantly lower body weights in mice housed in the IVC system (Pasquarelli et al. 2017). Kostomitsopoulos et al. Different types of ventilation systems (forced‐air IVCs and motor‐free IVCs) caused changes in mice weights; forced‐air IVCs had a lower weight but no significant difference in food intake (Kostomitsopoulos et al. 2012). In rats, forced‐air IVCs did not affect growth but showed significant differences in feed intake during the growing period (Kostomitsopoulos et al. 2011). Variables in noise and vibration in the habitat may affect weight gain, but further investigation is needed to clarify these changes.

In our study, we found that mice housed in IVC systems had higher body temperatures compared to those housed in OTC systems. We did not observe any differences depending on the type of groups (EG and NG). We also did not find any interaction between cage systems and group types. Takao et al. in 2016 found that stress affects body temperature (Takao et al. 2016). In their study with 3 different inbred strains, Åhlgren et al. found that mice housed in OTC had higher body temperatures compared to IVC (Åhlgren and Voikar 2019). In this study, housing conditions were examined for a short period of time. However, our study differs from this study as it examined long‐term housing conditions. These findings may explain the parallel trends in body temperature and weight gain, especially during early development. In our study, we also showed that mice housed in the IVC system had a time‐dependent increase in body weight and body temperature.

Studies have shown that different housing systems affect anxiety behavior in mice, with levels varying depending on sex and genetic background (Mineur and Crusio 2009; Åhlgren and Voikar 2019). Åhlgren et al. showed that the cage system did not significantly affect the behavioral responses of mice with different genotypes housed in OTC and IVC systems (Åhlgren and Voikar 2019). However, in mice with the same genotype, they found that mice housed in non‐motorized cage systems in EPM had lower anxiety behavior than those housed in IVC. This suggests that the air blowing and noise in the IVC system causes anxiety in animals (Polissidis et al. 2017). Given that differences between IVC systems may have different effects on animal behavior and research data, cage systems should be reported in more detail in research (Krohn et al. 2003; Krohn and Hansen 2010; Logge et al. 2013). Our study provides evidence that mice housed in IVCs exhibit more thigmotaxis behavior than mice housed in OTCs. These findings suggest higher levels of anxiety and stress in mice in the IVC group. Further research is needed to investigate the mechanisms underlying these findings and their potential impact on animal welfare and experimental outcomes.

OFT uses observation of the animal's movements and behavior in an open area, providing information about psychological states such as anxiety, exploratory behavior, and motor activity. Our findings from the OFT show that mice housed in IVCs showed significantly more entries into the outer zone and spent more time in this zone compared to mice housed in the OTC. However, there were no significant differences in entries into the inner zone, latency to enter any zone, overall activity levels, total movement distance, or speed. More outstanding entries and time spent in border zones may suggest a preference for peripheral zones, often associated with anxiety‐like behaviors (tigmotaxis), but the observed lack of significant changes in inner zone behavior or locomotor measures is notable. Our findings are in accordance with previous research highlighting the behavioral impact of cage systems. Pasquarelli et al. found that stress was significantly higher in mice housed in IVCs, suggesting that different housing systems have a significant impact on behavioral test results (Pasquarelli et al. 2017). Mineur and Crusio (2009) and Logge et al. (2013) reported that IVCs can increase anxiety‐like behaviors and alter drug sensitivity, with such effects varying by strain and sex (Mineur and Crusio 2009; Logge et al. 2013). Similarly, Polissidis et al. (2017) found that mice housed in different cage types exhibited different anxiety patterns in behavioral tests (Polissidis et al. 2017). These results highlight the importance of carefully selecting and reporting housing conditions in behavioral studies. Housing systems can introduce variability that can bias experimental results or affect reproducibility between laboratories. Given the increasing adoption of IVC systems in animal facilities, researchers should standardize and transparently document cage types and housing conditions when designing and performing behavioral experiments.

The EPM test measures anxiety levels in rodents by recording their behavior in a plus maze with open and closed arms (Polissidis et al. 2017). Rodents generally prefer closed arms, while those with anti‐anxiety behavior spend more time in open arms (Walf and Frye 2007). In our study, the EPM test area of the mice showed no difference between groups.

In our study, we examined the differences in pain responses in mice housed in different cage systems using nociceptive tests, tail‐flick, and hot‐plate tests. These tests measure acute thermal pain threshold responses, spinal responses, and complex peripheral pain behavior (Deuis et al. 2017; D'amour and Smith 1941). Our study is the first to examine the response of housing conditions (OTC and IVC) in BALB/c mouse strains to thermally painful stimuli using acute nociceptive tests. In contrast to the findings from the hot‐plate test, the results of the tail‐flick test suggest that housing conditions do not significantly affect spinal nociceptive reflexes in mice, as there was no statistically significant group effect. While the *p* value (*p* = 0.051) approached significance, it did not meet the conventional threshold, suggesting that the effect of environmental conditions may be more pronounced in supraspinal‐mediated responses, such as those assessed by the hot‐plate test, rather than in spinal reflex pathways. However, the time‐dependent increase in latency observed in both groups suggests a temporal modulation of pain sensitivity, possibly due to habituation, stress adaptation effects. These changes were more consistent and evident in the IVC group, where significant increases were observed at both time intervals. Such results may indicate that although housing does not directly affect baseline spinal responses, it may modulate nociceptive processing dynamics over time. The hot‐plate test revealed a significant main effect of housing condition, with mice housed in IVC showing delayed nociceptive responses at both 0 and 10 min. Notably, the sheltering effect was reduced at 20 min, which may reflect the adaptation effects of repeated thermal exposure. Within‐group analysis showed that both groups showed significant latency increases over time. The discrepancy between the hot‐plate and tail‐flick findings is in line with previous studies showing that different nociceptive tests activate different neural circuits (Casarrubea et al. 2011; Salberg et al. 2020; Segelcke et al. 2023). Hot‐plate responses involve higher brain centers, whereas tail‐flick responses are largely mediated at the spinal cord level (Barrot 2012; Segelcke et al. 2023). Therefore, the influence of environmental factors such as cage type may vary depending on the complexity of the pain pathway being assessed.

The results of the ELISA analysis indicated that the living conditions of the animals had a significant impact on their hormone levels, emphasizing how different cage types affect stress and metabolism. Specifically, the IVC and OTC cage systems caused different hormonal reactions related to ACTH, CORT, leptin, and ghrelin levels. The studies reveal that housing conditions, procedures, and unpredictable factors significantly increase serum ACTH and CORT levels in mice (Holubová et al. 2016; Kinlein et al. 2019; Segelcke et al. 2023). ACTH levels were greatly influenced by the type of cage and the experimental condition (EG and NG); mice in IVCs had higher ACTH levels than those in OTCs. Additionally, in both cage types, the EG groups had higher ACTH levels than the NG groups. Moreover, EG groups exhibited elevated ACTH levels in both cage types compared to NG groups. These findings align with previous research indicating that mice housed in IVC cages may elevate stress markers (Holubová et al. 2016; Kinlein et al. 2019).

CORT levels were affected mainly by the type of cage the mice lived in, with those in IVCs having higher CORT levels than those in OTCs, no matter the group they belonged to. The lack of a significant group effect shows that the long‐term living conditions have a more significant impact on regular CORT release than the short‐term testing methods used in this study. This evidence further supports the idea that cage type is a key factor affecting baseline stress physiology (Burman et al. 2014; Monteiro et al. 2015; Holubová et al. 2016; Takao et al. 2016; Kinlein et al. 2019). This study is the first to measure serum ACTH and CORT levels in mice with different cage systems, but further hormonal studies are needed to provide accurate information.

The hypothalamic‐pituitary‐adrenal (HPA) axis is a central component of the neuroendocrine system that regulates stress responses. Its dysregulation has been related to various physiological changes, particularly in the secretion of key hormones such as ACTH and CORT. Increased HPA axis activity affects appetite‐regulating hormones, especially leptin and ghrelin, which have emerging roles in stress regulation. (Finger et al. 2012b; Spencer et al. 2012; Monteiro et al. 2015; Holubová et al. 2016; Abizaid 2019).

Both the cage system and the group, as well as their interaction, significantly influenced leptin levels. In OTC‐housed mice, the EG group significantly increased leptin levels, whereas in IVC‐housed mice, leptin concentrations did not differ between the EG and NG groups. Interestingly, leptin levels were lower in IVC‐EG mice than in OTC‐EG mice, which might show differences in how energy is used or how temperature is managed in the two cage systems. Holubova et al. also showed in their 2016 study that leptin levels change with stress, and leptin levels are lower in stressed animals (Holubová et al. 2016). The hormone leptin has also been shown to influence stress responses by regulating stress‐related feeding behaviors (Pinto et al. 2004). The study examined the antidepressant‐like effects of the leptin hormone in an animal model of chronic stress. The hippocampus, a brain region that transduces leptin's antidepressant activity, was found to be a new paradigm for treating depressive disorders (Lu et al. 2006). It demonstrates that the study procedures spread out over time increase leptin levels and decrease the need for food intake due to the stress they cause, much like the effects of ghrelin. It shows mechanistically that chronically applied procedures create a chronic stress effect and a decreasing leptin relationship in the reflections of animal weights in the housing‐stress‐metabolism relationship. Ghrelin levels were much higher in mice in IVC cages in both groups, indicating that the cage type might influence hunger signals or energy balance. Even though the difference in ghrelin levels between the groups was only slightly significant, the steady increase in ghrelin in IVC mice supports the idea that living conditions change how the body controls eating behavior. Notably, unlike leptin, ghrelin levels were unaffected by the cage system and group interaction, suggesting a more generalized response to the housing environment.

Ghrelin, a hormone found in mammals, increases with stress in animals, potentially related to anxiety levels. This unexpected change in ghrelin levels occurs as part of the process of overcoming stress. Exogenously administered ghrelin stimulates ACTH release from the anterior pituitary and reduces glucocorticoid negative feedback in response to stress (Carlini et al. 2004; Spencer et al. 2012). Oversecretion of glucocorticoids can lead to memory loss, hippocampal atrophy, and excessive weight gain. This dysregulation of the HPA axis predisposes to anxiety. Ghrelin's function to regulate the HPA axis may induce a stress response and accelerate anxiety disorders with impaired ghrelin stimulation (Spencer et al. 2015, 2012). Ghrelin levels increase with stress, leading to increased food intake (Chuang et al. 2011). Ghrelin caused an increase in caloric intake in mice fed a low‐fat diet and exposed to stress (Finger et al. 2012a). Chronically stressed mice lost fat mass and reduced adiposity, while stress‐induced weight gain increased (Finger et al. 2012b). Our study results indicate that the increased ghrelin level in the housing‐stress‐metabolism relationship mechanistically indicates the effects on animal weight.

Regarding the mechanism relationship, many mechanisms have been examined as stress parameters in these studies. In the scope of our study, ACTH, CORT, leptin, ghrelin, adrenal size, and behavior‐stress‐painful stimulus changes were shown to be related to housing‐stress. Antinociceptive functions of ghrelin have been found in previous studies (Guneli, Yavasoglu et al. 2007, Guneli et al. 2010; Spencer et al. 2015, 2012; Pasquarelli et al. 2017). In the results of this study, it can be suggested that housing in IVCs increases ghrelin levels; increased ghrelin levels cause weight gain in animals (Chuang et al. 2011), and in this context, the mechanism by which ghrelin is related to pain responses can be suggested.

We suggest that leptin, another metabolic hormone, may also be related to pain by the opposite mechanisms. Holubová et al. showed in their study in rats that heterotypic stress‐induced decreases in leptin levels (Holubová et al. 2016), thus, similar to the mechanisms we mentioned for ghrelin, stress‐induced decreases in leptin levels delayed pain responses. In previous studies, it was found that leptin administered exogenously to mice decreased acute nociceptive responses (hot‐plate) (Kutlu et al. 2003), suggesting that low leptin levels in our study may be related to this mechanism.

## 5 Animal Welfare Implications

The results of this study have important implications for the welfare of laboratory mice housed in different cage systems. The observed differences in body weight, behavior, and levels of responses to painful stimuli highlight the importance of considering housing conditions in experimental design and animal care. It is important to recognize that differences in cage systems, such as IVC and OTC, can affect animal welfare and experimental outcomes.

The increased exploratory behavior and high levels of anxiety observed in mice housed in IVCs raise concerns about the potential welfare challenges associated with this housing system. Higher stress levels not only affect the reliability of experimental results but may also affect the overall welfare of the animals.

In addition, changes in metabolic hormones such as ghrelin and leptin suggest a complex interaction between housing conditions, stress and physiological responses. Stress‐induced changes in these hormones may contribute to changes in weight gain and potentially affect the long‐term health of the animals.

To improve animal welfare in laboratories, researchers and animal care staff should carefully consider the selection and standardization of cage systems. Detailed information about cage systems in research publications is essential for the scientific arena to correctly understand and interpret experimental results. Furthermore, continued efforts to improve housing conditions, minimize stressors, and emphasize animal welfare in research are critical to maintaining responsible and ethical animal research.

## 6 Conclusion

This study, evaluated as a whole, suggests that although there are different results in each part, the increase in body weight and body temperature in mice housed in IVC cages, the low levels of leptin relative to body weight, the increase in ghrelin, ACTH, and CORT levels, the high level of thigmotaxis in the OFT of anxiety tests, and the delayed response to stimuli in the hot‐plate suggest that the stress‐related welfare of the animals is low. This situation can be interpreted as the fact that the mice are isolated from the macroenvironment; they cannot hear or smell anything outside their small plastic boxes, there is constant airflow in the cage, and it is difficult to maintain a normal body temperature.

## Author Contributions

**Aslı Çelik**: conceptualization, methodology, investigation, formal analysis, data curation, visualization, writing – original draft, writing – review and editing, supervision. **Cemre Ural**: conceptualization, formal analysis, investigation, data curation, writing – original draft, writing – review and editing. **Hatice Efsun Kolatan**: formal analysis, data curation, investigation, writing – original draft, writing – review and editing. **Pembe Keskinoğlu**: methodology, writing – original draft, writing – review and editing, supervision. **Mehmet Ateş**: supervision, writing – original draft, writing – review and editing, methodology. **Zahide Çavdar**: writing – original draft, writing – review and editing, supervision, methodology. **Osman Yılmaz**: conceptualization, writing – original draft, writing – review and editing, supervision. **Mehmet Ensari Güneli**: conceptualization, methodology, writing – original draft, writing – review and editing, supervision.

## Ethics Statement

Approval was obtained from the ethics committee for animal experiments at Dokuz Eylul University Faculty of Medicine, Izmir‐Turkey (permission number, protocol number 08/2019). This article contains animals, and the ethics governing the use and conduct of experiments on animals were strictly observed.

## Conflicts of Interest

The authors declare no conflicts of interest.

### Peer Review

The peer review history for this article is available at https://publons.com/publon/10.1002/brb3.70601.

## Supporting information



Supporting information

## Data Availability

The data that support the findings of this study are available from the corresponding author upon reasonable request.
