# Supplementary material for: How Do Individually Ventilated Cages Affect the Welfare of Male BALB/c Mice? Comprehensive Assessment of Behavior, Metabolism, and Responses to Acute Painful Stimuli
Source: Brain Behav. 2025 May 30;15(6):e70601. doi: 10.1002/brb3.70601 (PMC12123442; doi:10.1002/brb3.70601)
Supplement: Supplementary file 1 — Supporting information [file BRB3-15-e70601-s001.docx]

**Supplement**

Table 2. Body temperature ranges across time points for different groups.The data shows the minimum-maximum values of body temperature measurements taken at five time points. n = 16 mice per group.

| **Time Point (P:Postnatal)** | **OTC-EG (°C)** | **OTC-NG (°C)** | **IVC-EG (°C)** | **IVC-NG (°C)** |
| --- | --- | --- | --- | --- |
| P58 | 36.3 - 37.3 | 36.5 - 37.8 | 37.2 - 38.0 | 36.8 - 38.8 |
| P66 | 36.1 - 37.4 | 36.2 - 37.8 | 37.3 - 38.4 | 36.7 - 38.2 |
| P76 | 36.0 - 37.1 | 36.0 - 37.5 | 37.3 - 38.4 | 36.9 - 38.6 |
| P86 | 36.0 - 37.8 | 36.0 - 37.8 | 36.9 - 38.0 | 37.1 - 38.3 |
| P103 | 36.1 - 37.1 | 36.1 - 37.9 | 37.2 - 38.5 | 37.7 - 38.9 |

**Number of fecal boli**

Fig. 8: Number of fecal boli in OFT and EPM areas. Statistical analysis was performed using the Mann-Whitney U test, which showed no significant difference between the groups (p > 0.05). Data are presented as mean ± SD for 16 mice per group.

**Tail-flick test**

Fig. 9: Tail-flick test responses of mice in the experimental groups. Statistical analysis was performed using the RM ANOVA test, which showed no significant difference between the groups (p > 0.05). Data are presented as mean ± SD for 16 mice per group.

**Adrenal size**

The study examined the impact of housing conditions and procedures on adrenal size in mice. Adrenal weights and adrenal weight/body weight ratios were analyzed in different groups (Table 3).

Table 3. Different cage systems and procedures significantly affected the adrenal weight of the mice. Two-way ANOVA analysis was performed to examine the effects of cage system (IVC vs. OTC) and group (EG vs. NG) on adrenal weight. Data are presented as means ± SEM. The number of subjects in each group was as follows: OTC-NG (n = 8), OTC-EG (n = 16), IVC-NG (n = 8), and IVC-EG (n = 16).

The analysis of left adrenal weights found a statistically significant effect of housing (OTC vs. IVC) (F_1, 44_ = 6.672, p = 0.013). The difference between groups (EG vs. NG) was not significant (F_1, 44_ = 0.185, p = 0.669). A significant interaction effect between housing and groups was found (F_1, 44_ = 6.162, p = 0.017).

A statistically significant effect of housing (OTC vs. IVC) was found in the analysis of right adrenal weights (F_1, 44_ = 13.537, p < 0.001). Groups (EG vs. NG) also had a significant effect (F_1, 44_ = 6.152, p = 0.017). A significant interaction effect between housing and groups was found (F_1, 44_ = 9.975, p = 0.003).

A statistically significant effect of housing (OTC vs. IVC) was found in the analysis of left and right adrenal weight means (F_1, 44_ = 12.691, p < 0.001). The difference between groups (EG vs. NG) was not significant (F_1, 44_ = 1.561, p = 0.218). A significant interaction effect of housing and between groups was found (F_1, 44_ = 10.239, p = 0.003).

| **Groups** | **n** | **Adrenal weights (mean±SD)** | | | |
| --- | --- | --- | --- | --- | --- |
|  |  | **Left (mg)** | **Right (mg)** | **Average (mg)** | **Adrenal weight/body weight ratio (mg/g)** |
| OTC-NG | 8 | 3.08 ± 0.42 | 2.46 ± 0.37 | 2.77 ± 0.35 | 0.10 ± 0.01 |
| OTC-EG | 16 | 3.36 ± 0.41 | 3.31 ± 0.45 | 3.34 ± 0.41 | 0.08 ± 0.01 |
| IVC-NG | 8 | 3.77 ± 0.50 | 3.49 ± 0.58 | 3.63 ± 0.41 | 0.08 ± 0.01 |
| IVC-EG | 16 | 3.37 ± 0.47 | 3.39 ± 0.54 | 3.38 ± 0.45 | 0.09 ± 0.01 |
| p value (OTC vs. IVC) |  | 0.013 | < 0.001 | < 0.001 | 0.274 |
| p value (EG vs. NG) |  | 0.669 | 0.017 | 0.218 | 0.854 |
| p value (OTC vs. IVC*EG vs. NG) |  | 0.017 | 0.003 | 0.003 | < 0.001 |

There was no statistically significant effect of housing (OTC vs. IVC) in the analysis of the mean ratios (adrenal weight/body weight ratio) of left and right adrenal weights and the mean ratio of measurements in the P58-P105 age range (F_1, 44_ = 1.228, p = 0.274). The comparison between groups (EG vs. NG) was not significant (F_1, 44_ = 0.034, p = 0.854). A significant interaction effect of housing and between groups was found (F_1, 44_ = 19.647, p < 0.001).
